# Urinary Lead Concentration Is an Independent Predictor of Cancer Mortality in the U.S. General Population

**DOI:** 10.3389/fonc.2018.00242

**Published:** 2018-06-29

**Authors:** Sen Li, Jiaxin Wang, Biao Zhang, Yuan Liu, Tao Lu, Yuanyuan Shi, Guangliang Shan, Ling Dong

**Affiliations:** ^1^School of Life Sciences, Beijing University of Chinese Medicine, Beijing, China; ^2^Department of Physiology, LKS Faculty of Medicine, University of Hong Kong, Hong Kong, Hong Kong; ^3^Department of Epidemiology and Statistics, School of Basic Medicine, Peking Union Medical College, Institute of Basic Medical Sciences, Chinese Academy of Medical Sciences, Beijing, China; ^4^Department of Biostatistics and Bioinformatics, Winship Cancer Institute, Emory University, Atlanta, GA, United States

**Keywords:** urinary lead, cancer mortality, National Health and Nutrition Examination Survey, epidemiology, biomarker

## Abstract

Lead is a ubiquitous pollutant that constitutes an environmental hazard worldwide. Although lead has been known as a carcinogenic factor in animal models, its role in human carcinogenesis is still a topic of debate with limited epidemiological evidence. Moreover, the association between urinary lead, as the most non-invasive and accessible way for lead measurement in human, and cancer mortality in general population has never been explored. We addressed this subject using continuous National Health and Nutrition Examination Survey 1999–2010 data and its Mortality Follow-Up Study. Of 5,316 subjects in study population, 161 participants died due to cancer. Cancer-specific mortality was associated with urinary lead levels after multivariable adjustment. Kaplan–Meier survival curve and cubic regression spline analyses indicated that high concentration of urinary lead exhibited significant association with raised death rate of cancer. Despite the marked decrease in environmental lead levels over the past three decades, lead exposure is still the significant determinant of cancer mortality in general population in U.S., and quantification of urinary lead may serve as a non-invasive approach to facilitate biomarker discovery and clinical translational research.

## Introduction

Lead is a ubiquitous environmental pollutant with a history of pollution for approximately 2,500 years (1). Lead can occur naturally, but its environmental presence is mainly from mining or historical use in paint and gasoline (2). Based on National Health and Nutrition Examination Survey (NHANES) data, the percentage of U.S. adults with blood lead concentration higher than 20 µg/dL reduced from 15% in NHANES II (1976–1980) to 0.6% in NHANES III (1988–1994) (3). The decreased blood lead concentration during late 1900s is attributed by reduction and elimination of lead in gasoline (4), restricted use of lead-based paints, and removal of lead solder from food cans (5, 6). However, the half-life of bone lead could be as much as 48.6 years, suggesting long-term effects of lead exposure on health and health disparities (7). Lead-containing deteriorated paint and ceramic food vessel also make lead pollution a health problem (8). Moreover, removal of environmental heavy metals is with difficulty, because they cannot be chemically or biologically degraded, and are normally indestructible. Thus, lead constitutes an environmental hazard worldwide (9).

The chronic influences of lead exposure are still uncertain (3). Several epidemiological reports have linked high concentration of lead in human with a variety of diseases and disorders, including heart disease (10, 11), hypertension (12), renal function impairment (13), and cancer (14). Although lead has been known as a carcinogenic factor in animal models, its role in human carcinogenesis is still a topic of debate. Because of limited epidemiological evidence, the IARC Working Group categorized inorganic lead compounds as “probably” human carcinogens (group 2A) (15). Recently, several epidemiological studies have illustrated the association of blood lead concentration and death rate due to cancer in numerous populations, including U.S. adults (14, 16), lead-exposed workers in South Korea (17) and Australia (18). However, whether urinary lead, as the most non-invasive and accessible way for lead measurement in human, is associated with cancer mortality has never been explored in the general population. Here, we demonstrate a significant association between urinary lead concentration and cancer mortality using data from NHANES and its Mortality Follow-Up Study.

## Materials and Methods

### Study Population

Continuous NHANES is a nationwide complex survey to collect and evaluate health and nutrition condition of the non-institutional civilian U.S. population (19, 20, 21). The survey data are released by National Center for Health Statistics biannually for public use since 1999, and NHANES has been approved by National Health Statistics Institutional Review Board. In this study, data from six NHANES survey cycles (1999–2010) and the NHANES (1999–2010) publicly available Linked Mortality File were employed to study the association between urinary lead concentration and cancer-specific mortality. NHANES Linked Mortality File is a follow-up study, in which the NHANES data are linked with National Death Index death certificate records. Present analyses were restricted to participants with age ≥40 years at baseline according to previous publication (16). Of the 19,968 participants with age ≥40 years, 6,490 (32.5%) had urinary lead data at the time of the examination. Four participants were excluded due to the inadequate information of follow-up. We also excluded 1,170 sampling persons who had insufficient information for other variables, leading to a final population of 5,316 participants in this study (Figure S1 in Supplementary Material). The median length of follow-up was 66 months, and 667 all-cause deaths (401 males and 266 females) and 161 cancer-specific deaths (100 males and 61 females) were recorded during follow-up. Analyses involving nine urinary metals further excluded participants with data missing for any of these nine metals, leading to a study population of 3,757 adults (Figure S1 in Supplementary Material).

### Cancer-Specific Mortality

Deaths due to malignant neoplasm were identified according to the leading causes of death as indicated in the public-use linked mortality file, which is based on the International Statistical Classification of Diseases, Injuries, and Causes of Death (ICD-10) guidelines (ICD-10 codes C00-C97). Participants with no information on death were deemed as alive and censored at the end of follow-up (December 31, 2011). For analysis of cancer-related mortality, follow-ups for participants with other leading causes of death were censored at the age when they died.

### Urinary Metal Measurement

Urinary samples were collected from eligible participants, and analysis was performed at Centers for Disease Control and Prevention. Samples were loaded into inductively coupled plasma-mass spectrometry, and urinary barium (Ba), cadmium (Cd), cobalt (Co), cesium (Cs), molybdenum (Mo), lead (Pb), thallium (TI), tungsten (W), and uranium (U) were measured. The detailed methodology is available in NHANES Laboratory Procedures Manual (22). Beryllium (Be), antimony (Sb), and platinum (Pt) were not included in this study because their readouts from a substantial number of measurements were below the limit of detection. In the analysis using urinary lead levels as a continuous variable, urinary lead concentration was log transformed because of their skewed distribution.

### Other Variables

The associations between urinary lead concentration and all-cause or cancer-specific mortality were adjusted for a series of potential confounding factors: age in years at baseline examination (40–49, 50–59, 60–69, or ≥70), race (Non-Hispanic white, Non-Hispanic black or others), education (<high school, high school or >high school), poverty income ratio (PIR; <1, 1 ≤ PIR ≤ median or >median, where medians were computed based on PIR ≥1 for each of the six data cycles), body mass index (BMI; <25 or ≥25 kg/m^2^, where BMI ≥25 kg/m^2^ indicates overweight based on NIH health guidelines), smoking status (yes or no, based on the question “Have you smoked at least 100 cigarettes in your entire life?”), alcohol usage (yes or no, based on the question “In any one year, have you had at least 12 drinks of any type of alcoholic beverage?”), diabetes status (yes or no, based on the question “Have you ever been told by a doctor or health professional that you have diabetes or sugar diabetes?”), and hypertension status (yes or no, based on the question “Have you ever been told by a doctor or other health professional that you had hypertension, also called high blood pressure?”). Moreover, urinary creatinine concentration was log transformed, and adjusted as an independent variable to account for urine dilution as suggested by other studies (23–25). To identify the relationship between urinary lead and blood lead, the ratio of urinary lead concentration (μg/L) to urinary creatinine concentration (mg/dL) (correction for urine dilution) was log transformed and applied in the correlation analysis with log transformed urinary blood lead concentration (μg/dL) as suggested (26).

### Statistical Analysis

Specific sample weights for subsample with urinary multi-analyte profile are employed to account for the complex sampling design following the NHANES Analytic and Reporting Guidelines. The weighted characteristics were calculated based on overall data, and data stratified by urinary lead levels (≤0.40, 0.41–0.73, 0.74–1.26 and >1.26 μg/L) or mortality status. Possible statistical differences of various variables at different urinary lead quartiles were accessed using the Rao–Scott chi-square test. In this study, the term “mortality” generally refers to cumulative mortality (i.e., prevalence of death). In addition, we also calculated mortality rates per 1,000 person-years, where the number of person-years was calculated as the time between baseline examination and date of death or the termination date of the study (December 31, 2011) (27). The association between urinary lead concentration and cancer-specific death rate was examined by Cox proportional hazards regression using “PHREG” procedure. In analyses for all-cause and cancer-specific mortalities, interaction of urinary metal levels with follow-up time was incorporated to examine proportional hazard assumption, and this interaction was leaved in the model if the assumption was violated in order to model non-proportional hazards as suggested (28). By comparing the first quartile of urinary lead levels, the hazard ratio, 95% confidence intervals (CIs), and *P*_trend_ for the risk of cancer death rate in regard to urinary lead quartiles were calculated. In addition to Cox regression, Fine and Gray regression was performed to account for death from other causes as competing events (29). The direct adjusted Kaplan–Meyer curves were generated using “DIRADJ” option. To improve normality, urinary lead levels were log transformed in the analysis of dose–response relationship for urinary lead levels and cancer-specific death rate, which was examined by three-knot restricted cubic splines (RCSs) using publicly available SAS macro (30). Covariate-adjustment for Cox hazards regression was employed for RCS analysis. SAS 9.4 software (SAS Institute Inc., Cary, NC, USA) was used to perform statistical analyses. Correlation structure of nine urinary metals was produced in R using the corrplot package.

## Results

**Figure 1:**
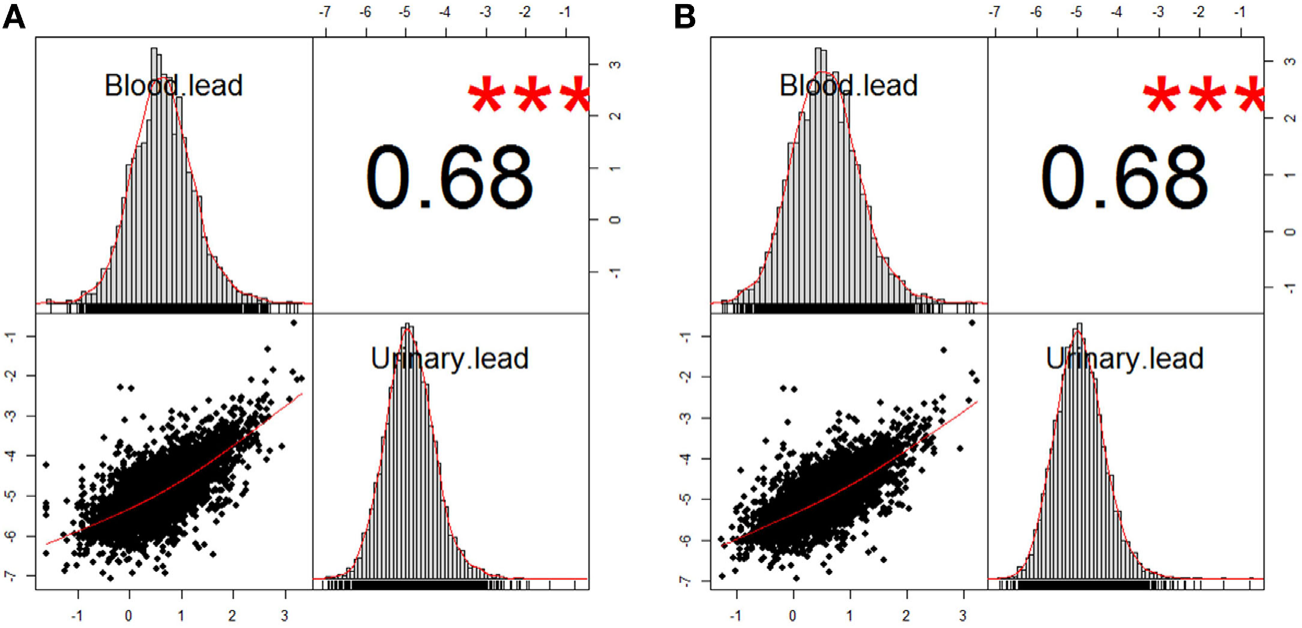
Correlation between blood lead and urinary lead in two analysis populations. **(A)** *n* = 5,193 after excluding participants with missing data of blood lead concentration from study population 1. **(B)** *n* = 3,677 after excluding participants with missing data of blood lead concentration from study population 2. The information about study population 1 and 2 can be found in Figure S1 in Supplementary Material. The correlation coefficients of both analysis populations were 0.68, and *** denotes *P* < 0.01.

Demographics of overall study population and subpopulations by urinary lead levels were demonstrated in Table 1. The sample size was 1,363, 1,307, 1,321, and 1,325 by urinary lead categories ≤0.40, 0.41–0.73, 0.74–1.26, and >1.26 μg/L, respectively, which was quartiles of distribution of urinary lead. Comparing to low urinary lead levels (≤0.40 μg/L), participants with high urinary lead (>1.26 μg/L) were more likely to be males, ≥70 years old, non-white ethnicity, to have less education, income below median, to be cigarette- and alcohol-users, and to present no history of hypertension. Moreover, urinary lead levels were correlated with all-cause mortality (*P* < 0.01). Among all the nine underlying causes of death included in NHANES mortality study, urinary lead levels were only significantly associated with cancer mortality (*P* < 0.01) (Table 1). In addition to cumulative mortality, we also calculated mortality rates for each quartile of the urinary lead concentration distribution. For all-cause mortality, mortality rates among participants with quartile 1, 2, 3, and 4 of urinary lead levels were 17.97, 19.45, 20.37, and 25.72 per 1,000 person-years, respectively. For cancer-specific mortality, mortality rates for quartile 1, 2, 3, and 4 of urinary lead levels were 2.89, 4.25, 5.35, and 7.44, respectively. It is of note that moderately strong positive relationship between urinary and blood lead concentration was revealed by correlation analyses in the analysis populations (Figure 1). We next investigated the possible correlation between mortality and other covariates and found that cancer mortality was correlated with age, education level, PIR and smoking status while all-cause mortality was associated with all the covariates listed in Table 2.

**Table 1:** Weighted characteristics of the study population by urinary lead level-NHANES 1999–2010.

					Urinary lead level (μg/L)												
		Overall			≤0.40			0.41–0.73			0.74–1.26			>1.26			
Variable	Status	*N*	%	SE	*N*	%	SE	*N*	%	SE	*N*	%	SE	*N*	%	SE	*P* value
Gender	Male	2,694	47.72	0.75	473	31.59	1.40	614	46.54	1.77	718	53.81	1.48	889	65.22	1.48	<0.01
	Female	2,622	52.28	0.75	890	68.41	1.40	693	53.46	1.77	603	46.19	1.48	436	34.78	1.48	

Age	40–49 years	1,452	35.38	1.04	421	38.43	1.92	344	34.47	1.69	366	36.14	1.77	321	31.18	2.05	0.05
	50–59 years	1,104	28.17	0.89	283	27.49	1.39	277	28.09	1.58	291	28.81	1.64	253	28.49	1.93	
	60–69 years	1,298	18.51	0.77	317	18.29	1.34	331	19.89	1.54	303	16.42	1.28	347	19.63	1.31	
	≥70 years	1,462	17.94	0.71	342	15.80	1.20	355	17.54	1.08	361	18.63	1.07	404	20.70	1.29	

Race	White	2,867	77.72	1.22	844	82.79	1.30	711	77.61	1.61	711	77.23	1.85	601	71.12	1.75	<0.01
	Black	1,024	9.75	0.72	172	6.08	0.64	252	10.07	0.89	275	10.50	1.02	325	13.75	1.27	
	Others	1,425	12.53	0.99	347	11.13	1.10	344	12.32	1.25	335	12.27	1.45	399	15.13	1.30	

Education	<High school	1,699	19.41	0.91	355	16.00	1.40	376	17.08	1.43	445	22.12	1.55	523	23.96	1.66	<0.01
	=High school	1,276	26.72	0.72	327	25.10	1.32	331	27.39	1.55	313	27.31	1.53	305	27.55	1.93	
	>High school	2,341	53.86	1.13	681	58.90	1.85	600	55.53	1.71	563	50.56	1.83	497	48.49	1.98	

PIR	<1	859	10.07	0.52	199	8.62	0.85	191	8.73	0.79	203	10.81	0.94	266	12.90	0.96	<0.01
	1 ≤ PIR ≤ median	1,988	29.08	0.89	477	27.05	1.57	475	28.46	1.42	511	30.28	1.79	525	31.35	1.71	
	>Median	2,469	60.85	1.14	687	64.32	1.89	641	62.82	1.63	607	58.91	1.92	534	55.74	2.01	

BMI	≥25	3,916	71.76	0.93	967	67.46	1.64	962	72.82	1.63	1,008	74.50	1.47	979	73.46	1.53	<0.01

Smoking	Yes	2,797	52.86	0.99	597	45.07	1.84	633	48.30	1.88	740	58.04	1.53	827	63.50	1.97	<0.01

Alcohol use	Yes	3,603	71.34	1.06	836	65.51	1.79	843	69.13	1.67	940	75.63	1.41	984	77.35	1.51	<0.01

Diabetes	Yes	821	10.76	0.45	231	10.77	0.95	217	12.20	0.98	204	10.68	0.85	169	9.09	0.89	0.17

Hypertension	Yes	2,380	39.07	0.99	664	42.42	1.88	621	39.98	1.73	570	37.51	1.55	525	34.98	1.72	0.01

Mortality	All-cause	667	9.01	0.50	137	7.62	0.70	142	7.68	0.79	160	8.86	0.87	228	12.79	1.20	<0.01
	CVD	145	1.80	0.20	31	1.64	0.37	38	2.14	0.41	33	1.65	0.36	43	1.79	0.35	0.73
	Cancer	161	2.35	0.23	22	1.62	0.39	31	1.76	0.47	42	2.53	0.48	66	3.89	0.60	<0.01
	CLRD	41	0.68	0.12	8	0.48	0.18	5	0.35	0.17	12	0.82	0.28	16	1.19	0.37	0.10
	Accidents	27	0.52	0.12	5	0.41	0.22	4	0.35	0.22	3	0.37	0.22	15	1.05	0.38	0.22
	CeVD	39	0.53	0.09	7	0.41	0.18	11	0.61	0.21	9	0.37	0.15	12	0.80	0.24	0.43
	AD	16	0.22	0.07	5	0.25	0.13	2	0.12	0.09	3	0.16	0.10	6	0.38	0.17	0.48
	Diabetes	21	0.20	0.05	5	0.31	0.14	4	0.12	0.06	6	0.17	0.08	6	0.20	0.10	0.49
	Flu & pneumonia	13	0.13	0.04	2	0.08	0.06	5	0.14	0.07	3	0.19	0.11	3	0.11	0.08	0.79
	Kidney disease	14	0.10	0.03	2	0.03	0.02	2	0.04	0.03	6	0.17	0.08	4	0.17	0.09	0.12

*N, number; %, weighted percent; NHANES, National Health and Nutrition Examination Survey; CVD, cardiovascular disease; CLRD, chronic lower respiratory diseases; CeVD, cerebrovascular disease; AD, Alzheimer’s disease; Flu & pneumonia, influenza and pneumonia; PIR, poverty income ratio; BMI, body mass index*.

**Table 2:** Weighted characteristics of the study population by mortality status-NHANES 1999–2010.

		All-cause mortality							Cancer mortality						
		Yes			No				Yes			No			
Variable	Status	*N*	%	SE	*N*	%	SE	*P* value	*N*	%	SE	*N*	%	SE	*P* value
Gender	Male	401	52.60	2.29	2,293	47.24	0.81	0.03	100	54.13	4.69	2,594	47.57	0.77	0.18
	Female	266	47.40	2.29	2,356	52.76	0.81		61	45.87	4.69	2,561	52.43	0.77	

Age	40–49 years	41	9.38	1.72	1,411	37.96	1.12	<0.01	13	9.82	3.35	1,439	36.00	1.06	<0.01
	50–59 years	64	17.58	2.25	1,040	29.22	0.95		19	22.58	4.58	1,085	28.30	0.91	
	60–69 years	138	19.03	1.91	1,160	18.46	0.80		44	27.47	4.98	1,254	18.29	0.78	
	>70 years	424	54.01	2.60	1,038	14.37	0.68		85	40.13	5.03	1,377	17.41	0.71	

Race	White	408	78.87	1.84	2,459	77.61	1.27	0.01	96	81.53	2.77	2,771	77.63	1.23	0.14
	Black	139	11.97	1.31	885	9.53	0.73		32	10.03	1.76	992	9.74	0.72	
	Others	120	9.16	1.54	1,305	12.87	1.01		33	8.44	1.98	1,392	12.63	1.00	

Education	<High school	299	37.01	2.71	1,400	17.67	0.86	<0.01	68	35.63	5.91	1,631	19.02	0.91	<0.01
	=High school	164	28.06	1.93	1,112	26.59	0.79		45	31.31	4.54	1,231	26.61	0.73	
	>High school	204	34.94	2.67	2,137	55.74	1.12		48	33.06	5.50	2,293	54.36	1.11	

PIR	<1	132	15.39	1.80	727	9.54	0.53	<0.01	23	9.07	1.79	836	10.09	0.52	<0.01
	1 ≤ PIR ≤ median	321	45.73	2.72	1,667	27.43	0.93		77	43.16	5.53	1,911	28.74	0.92	
	>Median	214	38.88	2.66	2,255	63.02	1.16		61	47.76	5.36	2,408	61.16	1.17	

BMI	≥25	440	65.61	2.42	3,476	72.37	1.00	<0.01	111	67.07	4.74	3,805	71.88	0.96	0.31

Smoking	Yes	410	64.54	1.97	2,387	51.71	1.05	<0.01	112	74.15	4.56	2,685	52.35	1.01	<0.01

Alcohol use	Yes	441	65.64	2.53	3,162	71.90	1.07	<0.01	122	77.70	4.62	3,481	71.19	1.05	0.19

Diabetes	Yes	160	19.92	1.77	661	9.85	0.46	<0.01	31	14.66	2.79	790	10.66	0.46	0.12

High blood pressure	Yes	370	54.08	2.70	2,010	37.58	1.11	<0.01	84	44.68	4.75	2,296	38.93	1.01	0.23

*N, number; %, weighted percent; NHANES, National Health and Nutrition Examination Survey; PIR, poverty income ratio; BMI, body mass index*.

**Figure 2:**
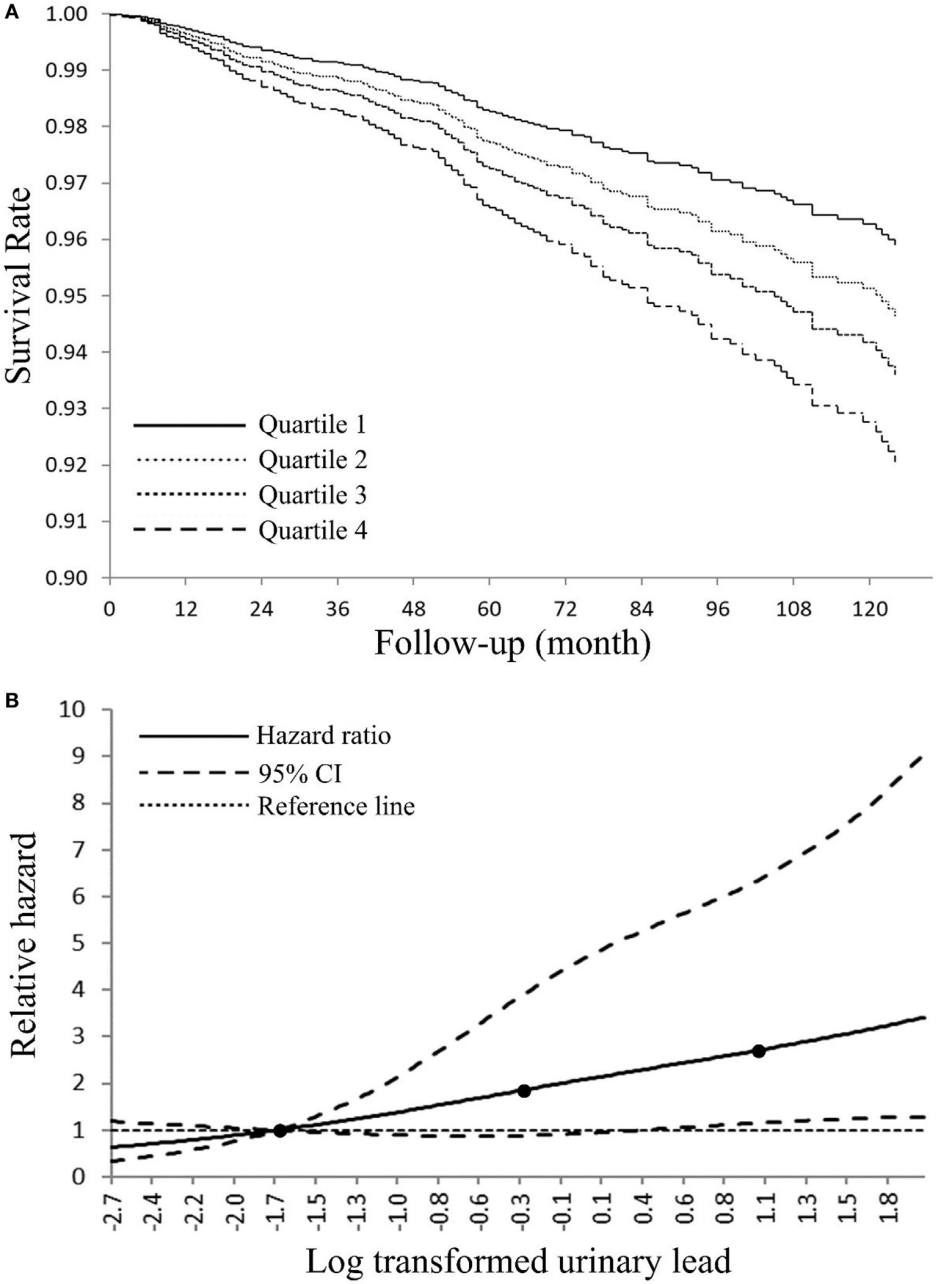
**(A)** Adjusted Kaplan–Meier survival curves for cumulative cancer mortality according to urinary lead quartile—National Health and Nutrition Examination Survey (NHANES) 1999–2010. Urinary lead quartiles: quartile 1: ≤0.40 μg/L; quartile 2: 0.41–0.73 µg/L; quartile 3: 0.74–1.26 µg/L; quartile 4: >1.26 μg/L. **(B)** Adjusted dose–response association between log transformed urinary lead and risk for cancer-related death-NHANES 1999–2010. Log transformed urinary lead was coded using a restricted cubic splines function with three knots (black dots) located at 5th, 50th, and 95th percentiles of its distribution. *Y*-axis represents the adjusted hazard ratio for cancer mortality for any value of log transformed urinary lead compared with a referent level of fifth percentile of its distribution.

We next calculated HRs from Cox models for all-cause and cancer-specific mortality by employing urinary lead quartiles as an independent variable (Table 3). For all-cause mortality, subjects in the highest urinary lead quartile exhibited a HR of 1.79 (95% CI = 1.15–2.78; *P* < 0.01), compared with participants with urinary lead below 0.40 µg/L after fully adjusting the covariates. Cancer-specific mortality was also associated with urinary lead levels after multivariable adjustment with adjusted HRs of 6.60 (95% CI = 2.37–18.37; *P* < 0.01). Trend analyses indicated significant results for the risk of both all-cause and cancer-specific mortality in regard to urinary lead levels (*P*_Trend_ < 0.01). Similar results were observed in a subsample excluding subjects who were diagnosed with cancer at baseline and die due to non-cancer causes (Table S1 in Supplementary Material). Furthermore, sex-stratified analysis indicated similar associations in both genders (Table S2 in Supplementary Material). In addition, analysis in a population of participants with age ≥20 years showed consistent results in Cox models (Table S3 in Supplementary Material). We next used the Fine–Gray competing risks method to estimate the association between urinary lead levels and cancer mortality after adjusting for covariates and accounting for potential bias caused by the competing risk of death from other causes, which indicated identical results (*P*_Trend_ < 0.01) with Cox models. In the overall study population (Figure S1 in Supplementary Material), 10-year survival was estimated by Kaplan–Meier curves for quintiles of the urinary lead (Figure 2A), and high levels of urinary lead were significantly associated with elevated mortality (*P*_Trend_ < 0.01). The dose–response relationship of urinary lead levels and cancer death rate was further studied in subjects with age over 40 years using log-transformed urinary lead concentration to model proportional hazards with a three-knot cubic regression spline (Figure 2B), which allows the shape of the relationship between the exposure and outcome to be flexible and not inherently linear. The result represented covariate-adjusted relative hazards with a referent level of fifth percentile of urinary lead distribution. The spline analysis showed that the relative hazards of cancer-specific mortality generally increased with urinary lead concentration, and lower bound of 95% CI of the relative hazard exceeded reference line (relative hazard = 1) at a urinary lead concentration of roughly 1.45 µg/L.

**Table 3:** Hazard ratio for all-cause and cancer mortality by urinary lead level-NHANES 1999–2010.

Urinary lead level (μg/L)	*N*	cHR (95% CI)	aHR (95% CI)^a^	aHR (95% CI)^b^
**All-cause mortality**				
≤0.40	137	1.00	1.00	1.00
0.41–0.73	142	1.21 (0.94–1.57)	1.59 (1.20–2.11)	1.22 (0.92–1.62)
0.74–1.26	160	1.40 (1.02–1.91)	2.05 (1.45–2.91)	1.40 (0.99–1.99)
>1.26	228	1.93 (1.32–2.83)	3.15 (2.05–4.83)	1.79 (1.15–2.78)
*P* for trend		<0.01	<0.01	<0.01
**Cancer mortality**				
≤0.40	22	1.00	1.00	1.00
0.41–0.73	31	2.32 (1.23–4.39)	2.79 (1.42–5.48)	2.05 (1.03–4.05)
0.74–1.26	42	4.26 (1.97–9.20)	5.52 (2.40–12.68)	3.68 (1.58–8.57)
>1.26	66	8.51 (3.42–21.16)	11.83 (4.38–31.93)	6.60 (2.37–18.37)
*P* for trend		<0.01	<0.01	<0.01

*cHR, crude hazard ratio; aHR, adjusted hazard ratio; CI, confidence interval; N, number of deaths; NHANES, National Health and Nutrition Examination Survey; BMI, body mass index; PIR, poverty income ratio*.

*^a^Model was adjusted for creatinine (a marker of urine dilution)*.

*^b^Model was adjusted for creatinine, sex, age, education, race, PIR, BMI, smoking status, alcohol-use status, diabetes status, and high blood pressure status*.

**Figure 3:**
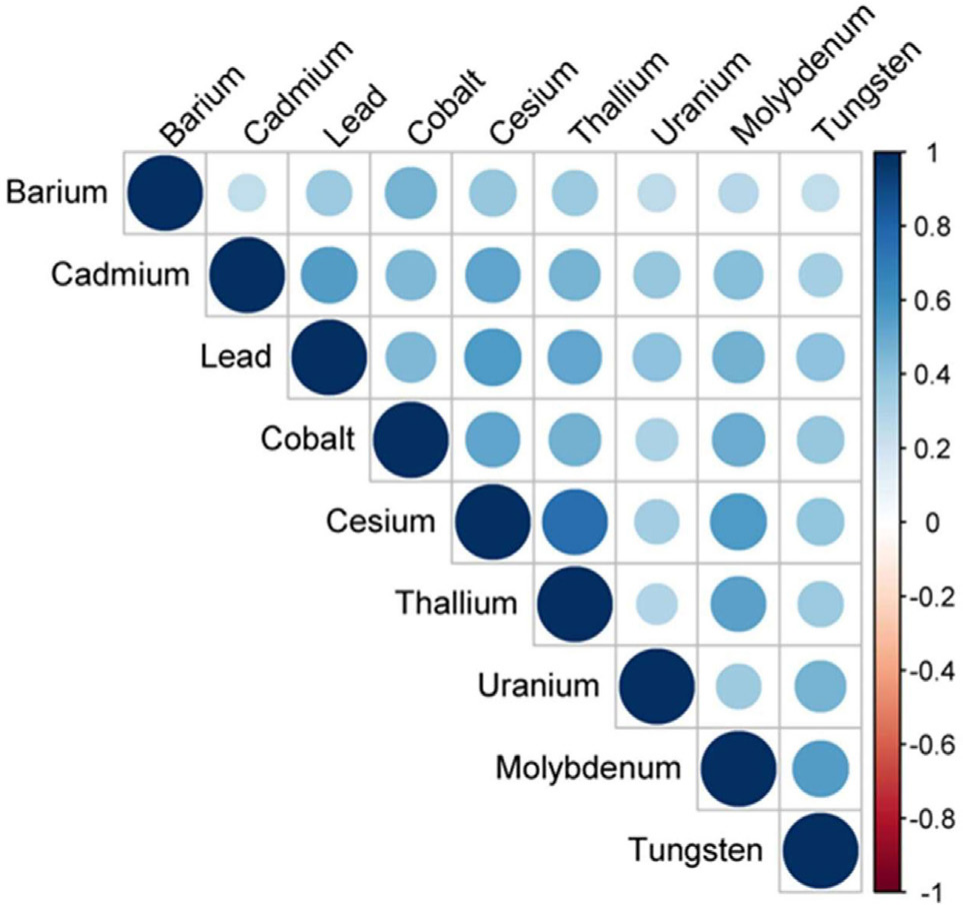
Correlation structure of nine urinary metals—National Health and Nutrition Examination Survey 1999–2010. Circle size and color intensity were in proportion to the correlation coefficients. The legend in the right side of correlation structure indicated the correlation coefficients and their corresponding colors. The nine urinary metals were ordered by hierarchical clustering.

**Figure 4:**
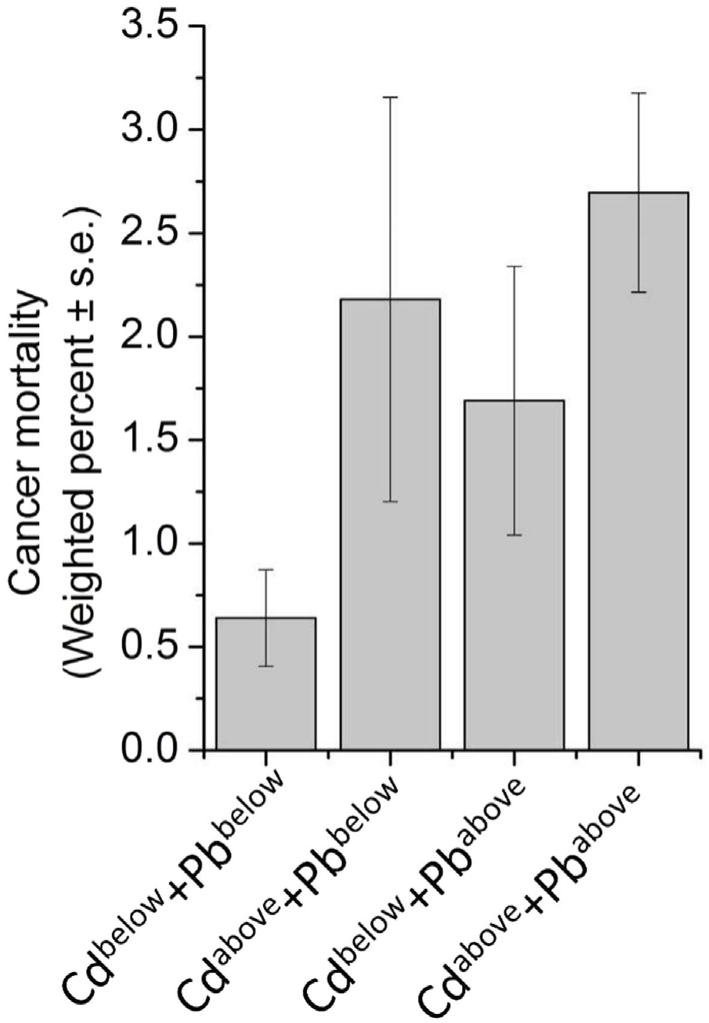
Weighted prevalence of cancer mortality according to Cadmium (Cd) and Lead (Pb) levels—National Health and Nutrition Examination Survey 1999–2010. For Cd, below the median: ≤0.350 μg/L; above the median: >0.351 μg/L. For Pb, below the median: ≤0.68 μg/L; above the median: >0.69 μg/L.

Considering that toxic metal exposure may occur with relatively high level of concordance, sensitivity analyses were performed for a panel of nine urinary metals in a subsample (Table 4). Besides the association between urinary lead levels and cancer mortality, our result further identified urinary cadmium levels as a risk factor (*P*_trend_ < 0.01) (Table 4). As expected, correlation analysis indicated that any pair of the nine metals was positively correlated (*P* < 0.01) (Figure 3). Due to the moderate positive relationship (*r* = 0.56) between urinary lead and cadmium, we next studied whether urinary lead levels could serve as a surrogate marker of urinary cadmium levels and were of no separate significance. Interestingly, urinary lead levels were persistently associated with cancer mortality after adjusting log transformed urinary cadmium concentration as confounder (*P*_trend_ < 0.05). In addition, a multiplicative interaction term between urinary cadmium and lead levels was entered into the main model, which showed non-significant result (*P*_interaction_ = 0.35). After categorizing urinary cadmium and lead above and below the median, relative risk (RR)-based analysis was performed, which indicated marginally increased RR for only cadmium above the median (RR_adjusted_ = 1.87; 95% CI = 0.74–4.71; *P* = 0.19) or only lead above the median (RR_adjusted_ = 1.78; 95% CI = 0.72–4.45; *P* = 0.22), but a significantly higher RR for cancer mortality in participants with both cadmium and lead above the median (RR_adjusted_ = 3.22; 95% CI = 1.41–7.35; *P* < 0.01) when setting participants with both cadmium and lead below median as reference. In consistent with this analysis, participants with only cadmium, only lead, or both metals above the median showed higher incidences of cancer mortality (2.18 ± 0.98, 1.69 ± 0.65, and 2.70 ± 0.48, respectively) (Figure 4).

**Table 4:** Hazard ratio for cancer mortality by level of nine urinary metals-NHANES 1999–2010.

Urinary metal level (μg/L)	*N*	cHR (95% CI)	aHR (95% CI)^a^	aHR (95% CI)^b^
**Barium**				
≤0.62	18	1.00	1.00	1.00
0.63–1.23	22	1.22 (0.65–2.27)	1.11 (0.59–2.09)	1.11 (0.59–2.10)
1.24–2.33	21	1.14 (0.61–2.13)	0.96 (0.50–1.85)	1.14 (0.59–2.22)
>2.33	16	0.87 (0.44–1.71)	0.70 (0.34–1.42)	0.79 (0.39–1.63)
*P* for trend		0.66	0.27	0.56

**Cadmium**				
≤0.180	11	1.00	1.00	1.00
0.181–0.350	11	0.97 (0.42–2.23)	1.13 (0.47–2.72)	0.88 (0.36–2.14)
0.351–0.627	14	1.23 (0.56–2.72)	1.55 (0.64–3.73)	1.13 (0.46–2.80)
>0.627	41	3.59 (1.85–6.99)	4.81 (2.09–11.08)	3.42 (1.36–8.58)
*P* for trend		<0.01	<0.01	<0.01

**Cobalt**				
≤0.200	12	1.00	1.00	1.00
0.201–0.324	21	1.88 (0.92–3.82)	1.83 (0.84–4.00)	1.68 (0.78–3.62)
0.325–0.501	21	1.85 (0.91–3.75)	1.79 (0.79–4.06)	1.61 (0.71–3.67)
>0.501	23	2.12 (1.05–4.25)	2.04 (0.87–4.79)	1.85 (0.79–4.33)
*P* for trend		0.05	0.19	0.25

**Cesium**				
≤2.88	11	1.00	1.00	1.00
2.89–4.68	25	2.40 (1.18–4.88)	2.06 (0.93–4.55)	1.74 (0.79–3.83)
4.69–6.94	24	2.25 (1.10–4.58)	1.80 (0.75–4.33)	1.63 (0.67–3.93)
>6.94	17	1.46 (0.68–3.11)	1.10 (0.41–2.98)	1.10 (0.40–3.07)
*P* for trend		0.52	0.56	0.75

**Molybdenum**				
≤22.5	12	1.00	1.00	1.00
22.6–41.1	20	1.70 (0.83–3.48)	1.62 (0.76–3.46)	1.42 (0.66–3.06)
41.2–69.2	24	2.02 (1.01–4.04)	1.87 (0.85–4.16)	1.83 (0.82–4.07)
>69.2	21	1.83 (0.90–3.72)	1.66 (0.70–3.94)	1.48 (0.63–3.51)
*P* for trend		0.09	0.32	0.41

**Lead**				
≤0.38	8	1.00	1.00	1.00
0.39–0.68	13	1.61 (0.67–3.88)	2.06 (0.80–5.29)	1.45 (0.57–3.71)
0.69–1.12	22	2.61 (1.16–5.86)	3.75 (1.45–9.69)	2.35 (0.91–6.10)
>1.12	34	3.70 (1.71–8.00)	5.74 (2.17–15.19)	3.15 (1.17–8.48)
*P* for trend		<0.01	<0.01	<0.01

**Thallium**				
≤0.086	20	1.00	1.00	1.00
0.087–0.146	23	1.19 (0.65–2.17)	0.78 (0.41–1.52)	0.87 (0.45–1.68)
0.147–0.226	17	0.86 (0.45–1.64)	0.45 (0.21–0.98)	0.61 (0.28–1.32)
>0.226	17	0.81 (0.42–1.54)	0.36 (0.16–0.84)	0.68 (0.29–1.57)
*P* for trend		0.35	<0.01	0.27

**Tungsten**				
≤0.033	18	1.00	1.00	1.00
0.034–0.070	24	1.29 (0.70–2.37)	1.10 (0.58–2.08)	1.12 (0.60–2.12)
0.071–0.131	19	1.23 (0.64–2.34)	0.98 (0.49–1.97)	0.95 (0.48–1.91)
>0.131	16	1.03 (0.52–2.02)	0.77 (0.36–1.64)	0.81 (0.38–1.74)
*P* for trend		0.96	0.42	0.49

**Uranium**				
≤0.0040	24	1.00	1.00	1.00
0.0041–0.0060	13	1.26 (0.64–2.47)	1.11 (0.55–2.23)	1.17 (0.58–2.36)
0.0061–0.0120	23	1.39 (0.79–2.47)	1.19 (0.64–2.19)	1.40 (0.75–2.62)
>0.0120	17	1.05 (0.56–1.96)	0.86 (0.43–1.70)	1.07 (0.52–2.18)
*P* for trend		0.67	0.77	0.68

*cHR, crude hazard ratio; aHR, adjusted hazard ratio; CI, confidence interval; N, number of deaths; NHANES, National Health and Nutrition Examination Survey; BMI, body mass index; PIR, poverty income ratio*.

*^a^Model was adjusted for creatinine (a marker of urine dilution)*.

*^b^Model was adjusted for creatinine, sex, age, education, race, PIR, BMI, smoking status, alcohol-use status, diabetes status, and high blood pressure status*.

## Discussion

Many metal ions are essential for human as trace elements, but they could be toxic at higher concentration. Lead is a ubiquitous environmental pollutant that can be found in lead-based paint and varnish, lead-using industries, lead solder, and leaded gasoline. Lead has various routes to enter the human body. For instance, lead particles from deteriorating lead-based paint or housing renovation adhere to food and therefore, can be ingested by humans. Industries that use lead in manufacturing can result in lead-polluted air and soil, thereby contaminating nearby animals and plants, which can transfer lead to humans *via* the food chain. Drinking water can also be contaminated by lead in plumbing solder. As a multitargeted toxicant, lead affects various systems, including cardiovascular, renal, and nervous systems with adverse impacts. The possible association between lead concentration in human and cancer-related death rate has also been reported in general population and occupational cohorts, although the conclusion is still plausible. Moreover, no previous work has investigated the relationship between urinary lead and cancer mortality while several studies have examined associations with blood lead using NHANES data that provide enough sociodemographic information to adjust for potential covariates. The analysis using NHANES II (1976–1980) data indicated that blood lead was a possible predictor of death rate (3). A NHANES III (1988–1994) mortality study suggested an association between blood lead concentration and risk of death from all causes and cancer among adults aged ≥40 years with blood lead concentration of 5–9 µg/dL (16). Consistently, Cheung reported the correlation between blood lead concentration and cancer mortality using NHANES III data (14). However, Menke et al. similarly using NHANES III data revealed that blood lead at substantially low levels (below 10 µg/dL) was correlated with elevated all-cause and cardiovascular death rate, but not cancer mortality (31). Inconsistent results were also reported in other general population-based studies. In the prospective Normative Aging Study, researchers showed bone lead levels, examined by K-shell X-ray fluorescence, were not correlated with cancer mortality, and were associated with a slight, but not significant, increase in all-cause and cardiovascular mortality (32). No association was found between blood lead concentration and cancer death rate in a cohort of 533 females enrolled in the study of Osteoporotic Fractures in U.S. from 1986 to 1988 (11). Because blood lead concentration of workers with occupational lead exposure is thought to be higher [often considerably over 80 µg/dL (33)] than that of general population (17), numerous occupational reports have examined the contribution of lead exposure to mortality of lead-exposed workers. However, the results from occupational studies are also controversial and inconclusive. In a cohort comprised 4,114 male lead workers, the all-cause mortality, mortalities due to liver or esophageal cancer were increased by lead exposure, as indicated by blood lead levels (18). Another cohort of 20,700 Finnish lead-exposed workers showed that high blood lead levels caused 1.4- and 1.8-fold raise of the overall cancer and lung cancer incidence, respectively (34). However, the effect of lead exposure on cancer mortality may be sex-specific as revealed by a cohort of 81,067 lead-exposed workers in South Korea, where cancer mortality was increased only in female workers (17). By comparing lead levels in blood, breast normal, and tumor tissues, clinical evidence suggested that lead exposure was an important risk factor for breast lesions (35), and urinary lead levels were increased in severe breast carcinomas (36). Our analyses employed the most up-to-date NHANES and its mortality data, and used urinary lead, as the most non-invasive indicator for lead content estimation in the human body. The results supported a correlation between urinary lead concentration and cancer death rate in general population.

Our data indicated that the weighted percent of men in low urinary lead quartile (≤0.40 μg/L) and high urinary lead quartile (>1.26 μg/L) are approximately 32 and 65%, respectively (Table 1). This is consistent with the idea that males are thought to have higher blood lead concentration than female in general because of higher lead exposure and blood hematocrit (8). Moreover, premenopausal women release bone lead more slowly than men, indicating a gender-specific discrepancy in lead metabolism (37). The gender-specific effect of lead on cancer mortality has been reported. Jemal et al. showed a significant spline dose–response result for high blood lead concentration (94th percentile) with a cancer mortality RRs of 2.4 compared with 12.5th percentile only in female population (38). However, we observed significant associations (*P*_trend_ < 0.05) between urinary lead levels and cancer-specific mortality in both genders in sex-stratified adjusted Cox regression (Table S2 in Supplementary Material). It is of note that HR estimate compared to the upper quartile may be unstable in the sex-stratified analysis because of few cancer deaths in the lowest quartile of urinary lead (*n* = 9 for male; *n* = 13 for female). Thus, most of our analyses combined data from both genders to increase statistical power.

Lead is expected to be a carcinogen in human according to occupational mortality and animal studies (16), and numerous mechanisms have been proposed for the carcinogenic effect of lead. One theory indicates the carcinogenic role of lead is through its capability to enhance cell proliferation (39). Experimental evidence shows that the proliferation of cultured bovine aortic smooth muscle cells can be induced by lead, but not other heavy metals such as zinc, copper, manganese, and nickel, and this stimulatory effect of lead on cell proliferation may be due to a calcium-dependent pathway considering that lead may mimic calcium in cellular metabolism (40). Moreover, lead has been reported to activate estrogen receptor-α and promote subsequent cell proliferation (41). Lead may also affect immune regulation and increase incidence of infectious diseases, autoimmune diseases, and cancer (42). By using cells isolated from lead-exposed individuals and unexposed healthy volunteers, Mishra et al. demonstrated that lead could target humoral and innate immune cells, thereby modulating immune system (42). Lead may also target various metalloproteins which represent around one-third of the proteome and serve as important regulators in physiological processes such as oxygen and electron transport and hydrolysis of amides and esters (43). Dysregulation of metalloprotein has also been known to associate with pathological events, including invasive breast cancers, and better understanding the role of metal ions may help to illustrate the metal function in human carcinogenesis (36). Indeed, it has been reported that lead is involved in carcinogenesis by modifying zinc level or replacing zinc in Zn-containing proteins (44). Indirect mechanisms of lead-related carcinogenesis such as production of free radicals and inhibition of DNA repair have been proposed (44). Siddiqui et al. reported that lead exposure induced oxidative damage caused by reactive oxygen species, thereby increasing the risk of breast lesions (35). As weak carcinogen, lead inhibits DNA repair and acts synergistically with other mutagens (45). It has been known that lead antagonizes selenium and minimizes its anti-carcinogenic effect, thereby increasing the risk for developing breast cancer (46). The effect of lead exposure on diseases may be subtle, and other genetic and environmental factors may also be involved (3). For instance, delta-aminolevulinic acid dehydratase (*ALAD*), a genitourinary cancer-related gene, is a target of lead. Inhibition of ALAD enzyme may diminish its role in heme biosynthesis and preventing protein degradation by 26S proteasome (44). Furthermore, the G177C genetic polymorphism of ALAD alters lead toxicokinetics and adverse effects of lead exposure, and ALAD^CG/CC^ genotype is associated with reduced death rate from all causes and from cancer (47). Thus, lead may promote carcinogenesis in human through multiple mechanisms.

Cancer is the second most common death inducer in many countries, and one of the most significant contributors to mortality worldwide. With the improvement in prevention and treatment of heart diseases, cancer is likely to become the number-one killer in the near further (48). Thus, detection early stage cancer is crucial to minimize tumor upstaging and increase patient survival rate, and prediction of further trends in cancer mortality may provide important implication for healthcare planning. Study of metal within a biological system may help people better understand the essential roles of metal in pathophysiology and provide a novel approach for disease detection and diagnosis (36). Indeed, urinary metal concentration has been served as biomarker for numerous physiological and pathological conditions in human, including thyroid function (49), renal dysfunction (50), and cardiac disease (51). Recently, an “omics” approach to simultaneously quantify 22 urinary metals (as metallomics) is established to facilitate personalized cancer screening and prevention, which reveals the significantly higher levels of lead in urine from breast cancer patients, indicating that urinary lead may serve as potential breast cancer biomarker (36). By employing NHANES and its mortality data, we reported 6.60-fold increase of cancer-specific mortality with high urinary lead levels (>1.26 μg/L) when comparing with those with low urinary lead levels (≤0.40 μg/L) (Table 3). This consistently introduces urinary lead as a non-invasive indicator to predict cancer-related mortality. Considering that lead exposure may co-occur with other toxic metals, we screened a panel of nine urinary metals and found any pair of these metals was positively correlated (*P* < 0.01) (Figure 3). Moreover, the association between urinary cadmium levels and cancer mortality was identified (*P*_trend_ < 0.01) (Table 4). Cadmium is a known human carcinogen as classified by IARC. Similar with lead, cadmium is also stored in bone (52). Due to its long biological half-life in human, urinary cadmium has been assumed as a biomarker for long-term cadmium exposure in various studies (53–56). More specifically, urinary cadmium is reported to be associated with cancer-caused mortality in general U.S. population enrolled in NHANES III (1988–1994) (54, 57), and American Indians participated in the Strong Heart Study (1989–1991) (56). Numerous possible mechanisms of carcinogenesis induced by cadmium have been proposed. Cadmium has been reported to modulate cellular proliferation and induce apoptosis. Indirect effects of cadmium also lead to oxidative stress and DNA damage (58). Moreover, cadmium reduces the capability for gene replacement in cells, thereby increasing the level of genetic instability (59). Cadmium may also serve as potent metallohormone and promote hormone-dependent carcinogenesis (60). For urinary cadmium and lead, their correlation has been reported (61). Thus, we further adjusted log transformed urinary cadmium concentration as an independent variable to exclude the possibility that urinary lead levels serve as a surrogate marker of urinary cadmium levels and are of no separate significance. The results consistently showed that urinary lead levels were associated with cancer mortality (*P*_trend_ < 0.05), indicating urinary lead levels as an independent predictor of cancer mortality. Furthermore, RR-based measurement between urinary cadmium and lead on the additive scale suggests that participants with both cadmium and lead above the median showed a relatively higher risk for cancer mortality (Figure 4). However, these analyses were based on 77 deaths due to cancer, and further studies with larger sample size are needed to verify our findings.

There are three ways to estimate human lead content: bone lead, blood lead, and urinary lead. Human skeleton deposits 95% of absorbed lead and serves as an endogenous reservoir for years after lead exposure (11). Thus, bone lead levels may provide a better indicator of cumulative lead exposure due to its longer half-life (62). Blood lead levels have been widely applied to estimate human lead exposure (63) while urinary lead may also be used for the assessment of lead exposed occupationally (64, 65) or environmentally (66). Indeed, urinary lead levels have been reported to be associated with bone health (67), toxocariasis infection in children (68) and asthma (69). Considering that urinary lead concentration may be relatively unstable compared to blood lead concentration, correlation analyses were performed, and the results showed moderately strong positive relationship between urinary and blood lead concentration in the analysis populations (Figure 1). Moreover, urinary lead is deemed as the most non-invasive and accessible way for lead measurement in human and is thus employed in this study. Current study has several limitations. First, site-specific cancer mortality is not provided because information of mortality due to specific type of cancer is not publicly available in NHANES (1999–2010) linked mortality file in order to protect the confidentiality of the participants. Considering the few total cancer deaths in the lowest quartile of urinary lead (*n* = 22) (Table 3), it is unlikely to have enough statistical power to identify associations between urinary lead levels and individual subtypes of cancer mortality because numbers of deaths from these cancers will be smaller (56). Moreover, this study has not illustrated the effects of urinary lead levels on cancer incidence and cancer survival, both of which contribute to cancer mortality. In addition, 10-year survival was calculated in our analyses (Figure 2A), but very few subjects were expected to have a follow-up of 10 years or longer (the median follow-up was 66 months). Furthermore, it is difficult to differentiate the acute and chronic effect of lead in investigations that only employ blood/urinary lead concentrations to represent exposure (62). Exposure misclassification serves as another potential limitation of this study, in which a single spot urine sample was employed to classify lead exposure. Both smoking status and alcohol-use status were assessed with a single question that could not distinguish light smokers/drinkers from heavy ones, which might be potential information bias. Despite these limitations, our study has several strengths. NHANES collected numerous health exposures and outcomes under an extensive quality control, and the survey data are generalizable to non-institutional civilian U.S. population. By employing the most up-to-date NHANES and its mortality data, our analyses screened nine urinary metals, and verified the association between urinary cadmium levels and cancer mortality. Moreover, this is the first study to demonstrate urinary lead concentration as an independent predictor of cancer mortality in general population.

## Conclusion

A positive correlation between urinary lead levels and cancer mortality was observed in the U.S. general population. Although further investigations are needed to illustrate the mechanisms, findings from the present study suggest a potential role of lead in cancer mortality, and a comprehensive understanding of lead content may help to discover novel diagnostic, prognostic, and therapeutic approaches for malignant neoplasms. Understanding of lead exposure routes such as dietary intake and environmental exposure may provide useful information on prevention of lead exposure, and its adverse effects.

## Availability of Data and Material

The data used in this study are from NHANES 1999–2010 and corresponding Mortality Follow-Up Study. Data are publicly available and can be downloaded from NHANES website: http://www.cdc.gov/nchs/nhanes.htm.

## Ethics Statement

Data analyzed in this study were obtained from NHANES. Protocols involved were approved by the National Center for Health Statistics (NCHS) Research Ethics Review Board (ERB), and consent from all participants was documented.

## Author Contributions

SL designed the study and drafted the manuscript. SL, JW, and BZ carried out the statistical analysis. YL, TL, YS, GS, and LD critically reviewed the manuscript. All the authors read and approved the final manuscript.

## Conflict of Interest Statement

The authors declare that the research was conducted in the absence of any commercial or financial relationships that could be construed as a potential conflict of interest.

## Abbreviations

NHANES, National Health and Nutrition Examination Survey; ICD-10, International Statistical Classification of Diseases, Injuries, and Causes of Death; NCHS, National Center for Health Statistics; NDI, National Death Index; ICP-MS, inductively coupled plasma-mass spectrometry; PIR, poverty income ratio; BMI, body mass index; cHR, crude hazard ratio; aHR, adjusted hazard ratio; CI, confidence interval; ALAD, delta-aminolevulinic acid dehydratase; CVD, cardiovascular disease; CLRD, chronic lower respiratory diseases; CeVD, cerebrovascular disease; AD, Alzheimer’s disease; Flu & pneumonia, influenza and pneumonia.
