# Supplementary material for: Urinary Lead Concentration Is an Independent Predictor of Cancer Mortality in the U.S. General Population
Source: Front Oncol. 2018 Jun 29;8:242. doi: 10.3389/fonc.2018.00242 (PMC6036403; doi:10.3389/fonc.2018.00242)
Supplement: Supplementary file 1 [file data_sheet_1.PDF]

Figures

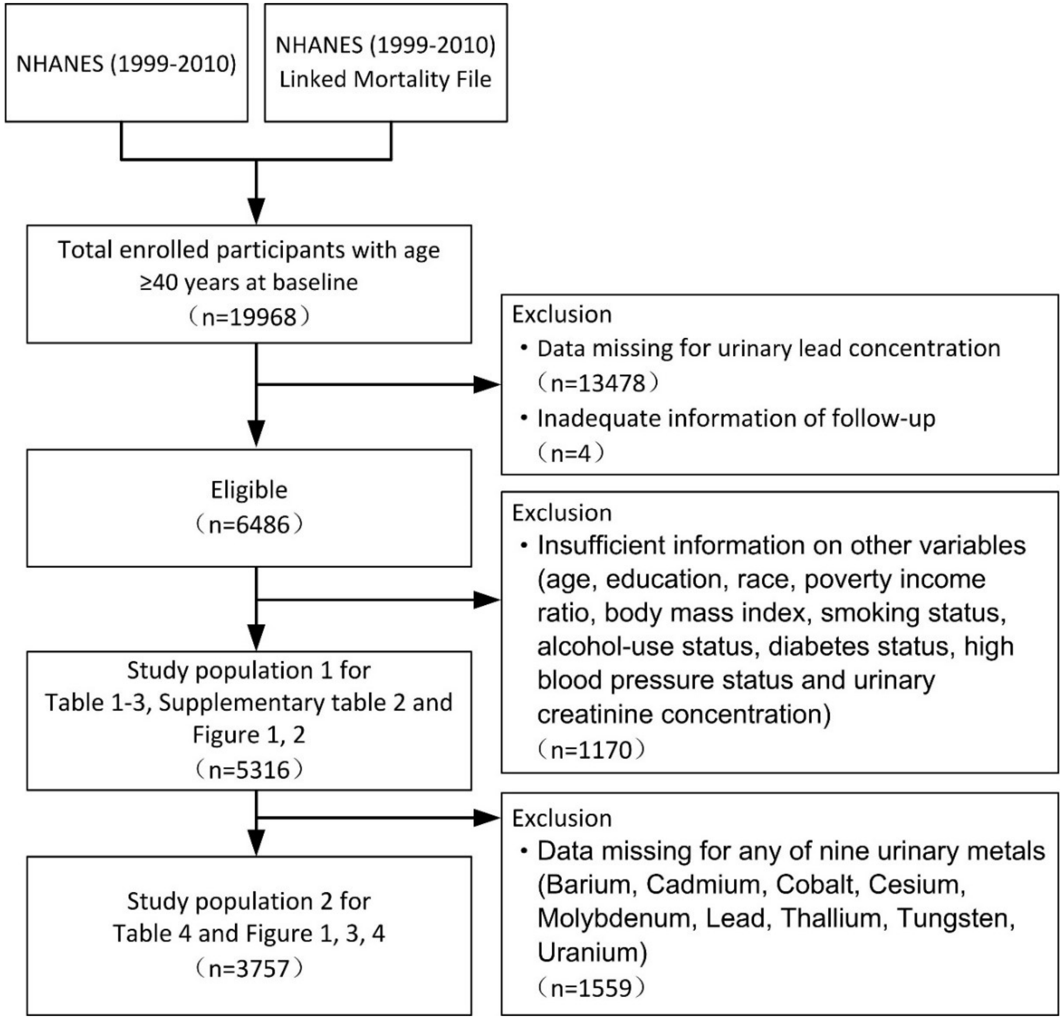

Supplementary figure 1. Participant enrollment flowchart including exclusion criteria-NHANES 1999-2010.

Supplementary table 1. Hazard ratio for cancer mortality by urinary lead level in a subsample (n=5211)-NHANES 1999-2010.

Urinary lead level (μg/L)	N	cHR(95% CI)	aHR(95% CI)[a]	aHR(95% CI)[b]
Cancer mortality				
≤0.40	22	1.00	1.00	1.00
0.41-0.73	31	2.33(1.23-4.40)	2.81(1.44-5.52)	2.05(1.04-4.05)
0.74-1.26	42	4.29(1.99-9.29)	5.62(2.44-12.91)	3.74(1.61-8.71)
>1.26	66	8.63(3.47-21.46)	12.11(4.49-32.68)	6.85(2.47-19.02)
P for trend		<0.01	<0.01	<0.01

Abbreviations: cHR, Crude Hazard ratio; aHR, Adjusted Hazard ratio; CI, confidence interval; N, Number of deaths; NHANES, National Health and Nutrition Examination Survey.
[a] Model was adjusted for creatinine (a marker of urine dilution).
[b] Model was adjusted for creatinine, sex, age, education, race, poverty income ratio, body mass index, smoking status, alcohol-use status, diabetes status, and high blood pressure status.

Supplementary table 2. Hazard ratio for cancer mortality by urinary lead level and sex category-NHANES 1999-2010.

	Male			Female		
Urinary lead level (µg/L)	N	aHR(95% CI)[a]	*P* value	N	aHR(95% CI)[a]	*P* value
≤0.40	9	1.00		13	1.00	
0.41-0.73	18	2.67(1.01-7.08)	0.05	13	1.43(0.53-3.85)	0.48
0.74-1.26	27	5.05(1.56-16.29)	<0.01	15	2.22(0.61-8.06)	0.23
>1.26	46	8.66(2.13-35.16)	<0.01	20	5.50(1.16-26.08)	0.03
P for trend			<0.01			0.02

Abbreviations: aHR, Adjusted Hazard ratio; CI, confidence interval; N, Number of deaths; NHANES, National Health and Nutrition Examination Survey.

[a] Model was adjusted for creatinine, age, education, race, poverty income ratio, body mass index, smoking status, alcohol-use status, diabetes status, and high blood pressure status.

Supplementary table 3. Hazard ratio for cancer mortality by urinary lead level in participants with age ≥20 years (n=8127)-NHANES 1999-2010.

Urinary lead level (μg/L)	N	cHR(95% CI)	aHR(95% CI)[a]	aHR(95% CI)[b]
Cancer mortality				
≤0.38	17	1.00	1.00	1.00
0.39-0.68	28	2.29(1.16-4.51)	3.48(1.72-7.03)	2.01(0.98-4.14)
0.69-1.14	46	5.11(2.30-11.36)	9.21(3.95-21.50)	3.91(1.63-9.39)
>1.14	72	10.35(4.01-26.68)	21.18(7.74-58.00)	6.55(2.27-18.91)
P for trend		<0.01	<0.01	<0.01

Abbreviations: cHR, Crude Hazard ratio; aHR, Adjusted Hazard ratio; CI, confidence interval; N, Number of deaths; NHANES, National Health and Nutrition Examination Survey.
[a] Model was adjusted for creatinine (a marker of urine dilution).
[b] Model was adjusted for creatinine, sex, age, education, race, poverty income ratio, body mass index, smoking status, alcohol-use status, diabetes status, and high blood pressure status.
